# malERA: An updated research agenda for basic science and enabling technologies in malaria elimination and eradication

**DOI:** 10.1371/journal.pmed.1002451

**Published:** 2017-11-30

**Authors:** The malERA Refresh Consultative Panel on Basic Science and Enabling Technologies

## Abstract

Basic science holds enormous power for revealing the biological mechanisms of disease and, in turn, paving the way toward new, effective interventions. Recognizing this power, the 2011 Research Agenda for Malaria Eradication included key priorities in fundamental research that, if attained, could help accelerate progress toward disease elimination and eradication. The Malaria Eradication Research Agenda (malERA) Consultative Panel on Basic Science and Enabling Technologies reviewed the progress, continuing challenges, and major opportunities for future research. The recommendations come from a literature of published and unpublished materials and the deliberations of the malERA Refresh Consultative Panel. These areas span multiple aspects of the *Plasmodium* life cycle in both the human host and the *Anopheles* vector and include critical, unanswered questions about parasite transmission, human infection in the liver, asexual-stage biology, and malaria persistence. We believe an integrated approach encompassing human immunology, parasitology, and entomology, and harnessing new and emerging biomedical technologies offers the best path toward addressing these questions and, ultimately, lowering the worldwide burden of malaria.

## Abstract 2

Dyann F. Wirth and colleagues propose a research agenda for basic science and enabling technologies in malaria elimination and eradication.

**Summary points**

- The recent development of multiple in vitro systems for studying malaria biology has helped deepen our understanding of the disease. Nevertheless, research remains hampered by a lack of in vitro models that can probe key aspects of malaria (e.g., gametocyte development in *Plasmodium vivax*, fertilization, ookinete biology, parasite–midgut interactions, human hepatocyte infection) and generate biological materials (i.e., infectious sporozoites) for laboratory study. Developing the necessary cell lines and other in vitro culture tools to propel these studies represent important areas for future research.
- With the emergence of widespread insecticide resistance in mosquito populations, there is a strong need to bring basic research in mosquito biology back into the malaria eradication agenda to strengthen current insecticide-based control campaigns and generate alternate vector control strategies.
- Driven by the development and accessibility of large-scale research tools and technologies, the scientific community can systematically tackle key questions in malaria, such as the following. What are the genes that contribute to antimalarial drug resistance (thereby defining the full parasite “resistome”)? What are the functions of key *Plasmodium* genes (providing much-needed annotation of key *Plasmodium* genes)? What are the genes and gene mutations that drive resistance in mosquito populations?
- Continued exploration of the potential of enabling technologies is needed. Important areas of future research include the use of gene-drive strategies and other gene-manipulation technologies; metabolomics-based approaches for biomarker discovery; structural vaccinology, novel technology platforms, and the use of novel adjuvants to improve vaccine design; and high-throughput approaches to facilitate drug discovery and screening.

## Background

Since the first agenda for malaria eradication was published in 2011 [1], there have been many significant developments in basic science, including an enhanced understanding of parasite biology (both gametocyte and liver stages) as well as mosquito biology (Table 1). Some of these advances could not have been predicted 5 years ago, such as the use of mouse models engrafted with human liver to advance the biology of liver-stage parasites (including the quiescent *P*. *vivax* hypnozoite stage) and the development of powerful genome-editing capabilities based on clustered regularly interspaced short palindromic repeats/associated protein-9 nuclease (CRISPR/Cas9) technology. In contrast, little progress has been achieved in several key research areas that were previously prioritized and, as such, they remain important stumbling blocks on the road to eradication.

We focus here on these and other crucial areas—deficiencies in basic science research and the lack of enabling technologies—that currently limit our progress towards malaria elimination and eradication. Importantly, this analysis highlights specific aspects of the *Plasmodium* life cycle in both the human host and the *Anopheles* vector. Our integrated approach aims to combine research efforts and expertise across human immunology, parasitology, and entomology to introduce powerful new ideas and technologies from other fields, provide a multifaceted view of disease biology, and accelerate progress toward eradication.

**Table 1:** A listing of the important research areas highlighted in malERA 2011, the progress made since then, and the remaining areas that require additional research.

Research Area	Accomplishments in Past 5 years	References	Remaining Gaps
Transmission Biology (Gametocytes to Mosquito)	Improved understanding of transcriptional and epigenetic control of sexual development	[2–6]	Limited work on *P*. *vivax* gametocytes due to lack of in vitro culture system
	Drug screens targeting transmission stages	[7–11]	
	Improved understanding of mosquito host-seeking behavior and olfaction biology	[12–16]	
	Improved understanding of mosquito–parasite interactions	[17–20]	
	*Anopheles* midgut cell line model for in vitro ookinete production and invasion	[21–25]	
Infection Biology (Mosquito to Liver)	Humanized mouse model for entire life cycle of *Plasmodium*, including *P*. *vivax* hypnozoites and liver stages	[26, 27]	Methods to increase sporozoite availability
	In vitro models for *Plasmodium* liver stages	[28–30]	
	Genetic crosses in mouse model	[31]	
	Primate models for *P*. *cynomolgi*	[32]	
	Controlled human malaria infections with sporozoites and blood-stage parasites	[33–40], reviewed in [41]	
Biology of Blood-stage Parasites	Improved production of continuous culture conditions, including identification of host cell environments necessary to support *P*. *vivax* invasion in culture and proof-of-principle that human hematopoietic stem cells can be immortalized, expanded, and differentiated into reticulocytes	[42–55]	No in vitro culture system for *P*. *vivax* asexual stages has been developed Poor functional annotation of genes
	*P*. *knowlesi* in vitro culture adaptation	[56, 57]	
	Identification and spread of mutations associated with artemisinin resistance	[58–64] reviewed in [65–67]	
	Comparison of mitochondrial and lipid metabolism of *P*. *falciparum* in sexual and asexual blood stages	[68, 69]	
Persistence of Parasites and Mosquitoes	*P*. *vivax* hypnozoites cultured in vitro	[26, 28]	Biomarkers for asymptomatic hosts Ecology and migration rates of vector species Long-term behavioral resistance studies
	Mosquito dry season estivation and long-distance migration observed in sub-Sahelian populations	[70]	
	Mechanisms of insecticide resistance identified	[71–73]	
Additional Technological Developments	Mosquito genomic resources to identify population substructure and allow comparative genomic studies	[74–77]	Coordinated efforts to generate knockout or knockdown libraries to understand gene function, especially in human parasites
	Genome-editing systems (CRISPR/Cas9, Zinc-finger nuclease), posttranslational protein knockdown systems (DD tag, Riboswitch), conditional genome deletion systems (Cre-LoxP, FLP-frt, diCre), conditional gene expression system (TetR-aptamer)	[78–86]	
	Proofs-of-principle for population suppression and population modification/replacement of *Anopheles* using gene drives	[87–92]	
	Colonization of important mosquito vector species	[93]	
	New techniques to improve antigen design and clinical evaluation of vaccine candidates	[94–100]	
	Improved resolution in intravital imaging	[101, 102]	

**Abbreviations:** Cre-LoxP, genetic recombination system involving the Cre (Causes recombination) protein and *loxP* (locus of X-over P); CRISPR/Cas9, clustered regularly interspaced short palindromic repeats/associated protein-9 nuclease; diCre, dimerizable Cre recombinase; DD, destabilization domain; FLP-frt, Flipase used to recombine two frt domains; malERA, Malaria Eradication Research Agenda; TetR, tetracycline repressor.

## Methods

The findings presented in this paper result from an extensive literature review of published and unpublished materials and the deliberations of the 2015 Malaria Eradication Research Agenda (malERA) Refresh Consultative Panel on Basic Science and Enabling Technologies. Electronic databases were systematically searched for published literature between January 1, 2010, and July 2, 2016, without language limitations. Panelists were invited to recommend additional literature and additional ongoing research projects. A 2-day workshop was held with the majority of the panel members, including field researchers, specialists from basic science, malaria genomics and epigenomics, regenerative medicine, and National Institutes of Health representatives. The panel broke into 6 breakout sessions to identify the problems that need to be solved in asexual blood stages, liver stage and mosquito, mosquito, *P*. *vivax*, population genetics and resistance, and transmission. The panel discussed what research is needed to address these problems and considered 6 crosscutting themes in CRISPR technologies, immunology and malaria vaccines, genomics tools for malaria, metabolism and malaria, structural biology, and diagnostics for malaria. Each group fed back to plenary session, where further robust discussions and input occurred. This helped refine the opportunities and gap areas in which research is needed. The final findings were arrived at with inputs from all panelists and several iterations of the manuscript.

## Advances, challenges, and opportunities in transmission biology

### Gametocytes

**Fig 1:**
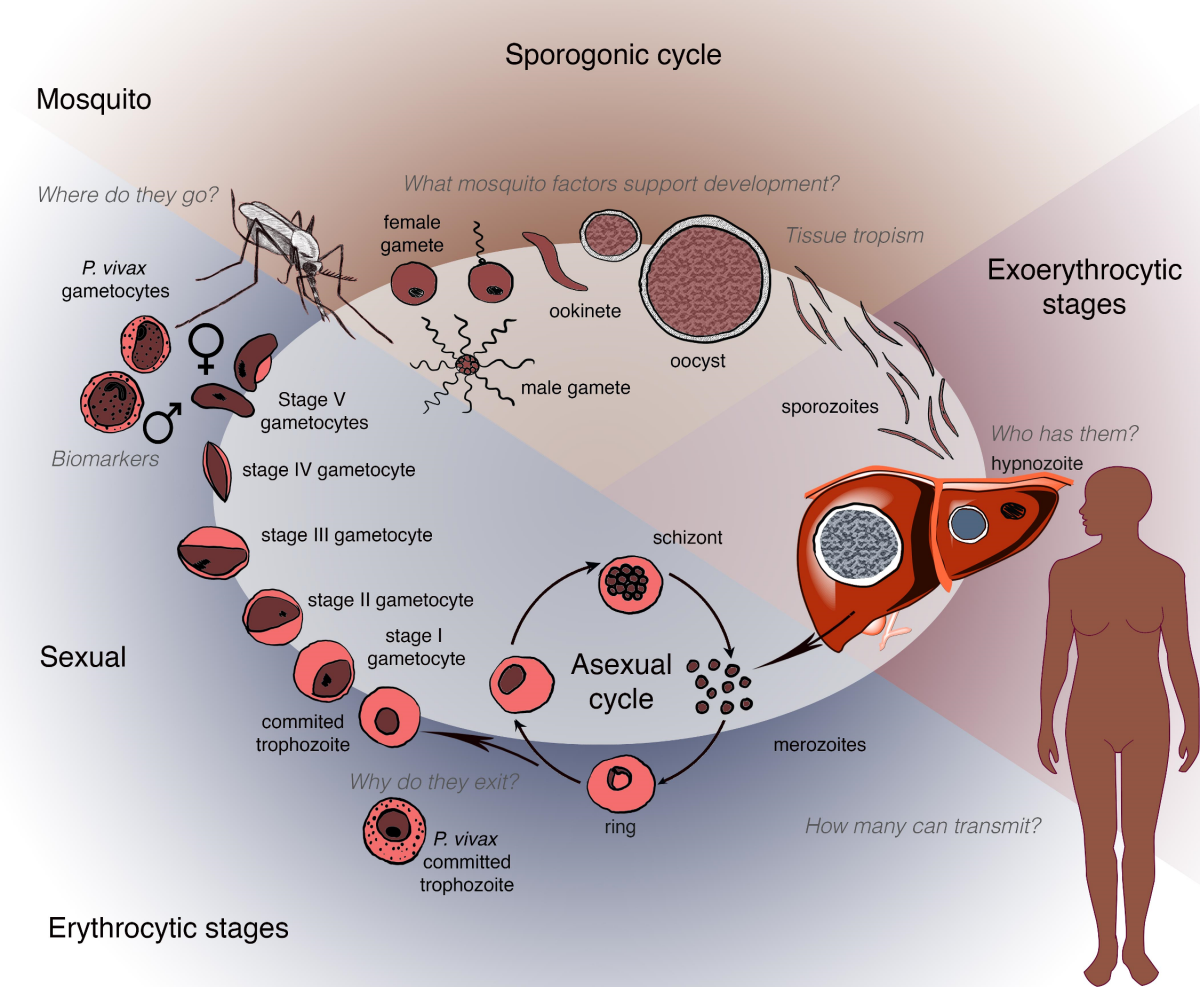
Schematic depicting the human and mosquito life cycles of *Plasmodium*, highlighting critical questions at specific points within the life cycle.

*Plasmodium* transmission begins with the development of sexual forms of the parasite (known as gametocytes) in an infected human host and their subsequent transfer to an anopheline mosquito following a blood meal (Fig 1). This stage represents a key bottleneck in the parasite life cycle and thus is an attractive opportunity for disrupting disease transmission. As shown in Box 1, in the past 5 years significant and exciting progress has been made in understanding gametocyte development, including insights into the transcriptional and epigenetic control of sexual differentiation and evidence for bone marrow sequestration [2–6, 103]. In the case of *P*. *falciparum*, newly available in vitro systems for gametocyte maturation have been used in small molecule screening, antibody reagent development, and transcriptional and metabolomics analyses [7–11].

**Box 1. Opportunities for the next 5 years**

1. **Functional genomics**
  - Identification of regulatory sequences within the parasite genome, similar to the human Encyclopedia Of DNA Elements (ENCODE) project,
  - Genome wide annotation of gene function in human parasites to identify sets of genes involved in discrete cellular processes, including drug resistance,
  - Improved scalability of CRISPR/Cas9 technology in asexual parasites to allow for both pooled, genome-wide approaches (large scale) and single cell transformation (microscale),
  - Greater collaboration between researchers to avoid overlapping gene annotation efforts.
2. **Advances in mosquito biology**
  - Generation of a mosquito consortium to evaluate promising gene drive-based strategies for efficacy at scale and/or over time and share knockout and/or transgenic strains,
  - Greater understanding of mosquito behavior and ecology,
  - Colonization of important vector species,
  - Development of in vitro mosquito infection models.
3. **New vaccine approaches**
  - Improved adjuvants and identification of new targets, including better structures for existing (and new) targets to improve structural approaches,
  - Development of novel approaches with the potential to generate sterilizing immunity (i.e., cognate antigens),
  - Coordinated functional annotation of asexual-stage parasites to enable prioritization of functional vaccine antigens,
  - Greater access to samples and data from both human challenge studies and patient samples demonstrating natural immunity,
  - Application of gene-editing technologies to systematically understand the function of hypothetical genes.
4. **Biomarkers and diagnostics**
  - Indicators of transmissible gametocytes,
  - Markers of liver-stage infection, in particular, hypnozoites,
  - Markers/assays to identify asymptomatic carriers,
  - Identification of metabolic signatures of different stages of the life cycle.
5. **Greater understanding of resistance to antimalarials and insecticides**
  - Identification of genes and pathways (i.e., the “resistome”) involved in resistance,
  - Development of alternatives to insecticides,
  - Use evolutionary approaches to prevent resistance.
6. **Greater accessibility to *P*. *vivax* gametocytes**
  - Development of a *P*. *vivax* in vitro culture system (e.g., ookinetes to validate transmission-blocking vaccine targets),
  - Greater collaboration between groups to improve access to existing sporozoite sources. This would be coupled with advances in cryopreservation to improve access to sporozoites globally.

In contrast, the mechanisms of *P*. *vivax* gametocyte development remain largely unknown. Gametocyte biology within this species is quite distinct—development takes just 2 to 3 days and unfolds prior to any clinical symptom. *P*. *vivax* gametocytes appear susceptible to existing antimalarial drugs that are not effective against *P*. *falciparum* gametocyte stages [104–106]. Progress in this area has been hampered by the absence of a comparable in vitro culture system for asexual *P*. *vivax* parasites, which is an urgent priority, as it would enable the generation of gametocytes for laboratory study, mosquito infections, and sporozoite production.

Another major area for discovery is the elucidation of the biological determinants of gametocyte transmissibility, especially in areas of low endemicity. Does the success of transmission depend on gametocyte quantity and/or quality? Are there mosquito-specific factors that actively recruit gametocytes to the biting site or do gametocytes preferentially sequester near the skin? What factors and mechanisms enable male and female gametes to find one another in the mosquito midgut? Biomarkers for transmission competency could enable a broader understanding of the heterogeneity in natural infections.

### Mosquito biology and host seeking

Transmission success also depends upon the interactions of the mosquito vector with both its human host and ingested parasites. Since 2011, there have been major advances in understanding the biology of olfaction and host-seeking behavior in mosquitoes via a combination of behavioral assays, electrophysiology, and functional genomic approaches [12–16]. High-throughput screens have identified new classes of attractants and repellents that are currently being tested in mosquito traps and spatial repellent trials ([107–110], also see MESA Track at http://www.malariaeradication.org/mesa-track). Moving forward, the identification of oviposition cues and the role of olfaction and taste in larval stages could facilitate the development of additional tools for vector control. Comparative genomic analysis of odorant receptor pathways that differ between anthropophilic and zoophilic species will help to elucidate the molecular basis of host-seeking behavior. Recent studies have shown that the composition of the human skin microbiota influences host attractiveness to mosquitoes [111] and identified volatile substances produced by parasites in human hosts thought to preferentially attract mosquitoes to infected individuals [112]. Nevertheless, gaps remain in our knowledge regarding the potential for gametocyte-seeking behavior by the mosquito and parasite-induced changes to the human host that may influence mosquito behavior to enhance biting and transmission.

### Parasite development in the mosquito

Fertilized zygotes develop into the motile ookinete, which in turn crosses the midgut wall. Major advances have been made in understanding midgut invasion and early mosquito anti-*Plasmodium* immune responses that target the ookinete stage. Several parasite genes that interact with the vector to enable its invasion of epithelial cells have been identified [17–19], and new insights have emerged regarding the role of epithelial responses to invasion and the corresponding epithelial interactions with the complement-like system to limit ookinete survival [113–117]. There is increasing evidence that the oocyst stage is also a target of innate immunity in the mosquito [118, 119]. Genome-wide association study (GWAS) mapping of *Anopheles* populations displaying different vector competence has identified mosquito genes that influence parasite development [120]. This list of potential targets to disrupt malaria transmission could be extended through functional screens using double-stranded ribonucleic acid (dsRNA)-mediated gene silencing in mosquitoes and synthetic approaches such as single-chain antibodies to block *P*. *falciparum* from infecting salivary glands.

A particular challenge for developing new interventions is the lack of culture systems to study fertilization, ookinete biology, and parasite–midgut interactions in human malaria parasites. *Plasmodium* species of rodents and birds have provided rapid proof-of-principle for new transmission-blocking strategies [121–123] and will likely continue to be critical for revealing the basic biology of sexual and mosquito stages. The development of mosquito midgut-derived cell lines (or organoids) supporting the in vitro culture of ookinetes and oocyst of human malaria parasites would enable high-throughput transcriptomic and metabolomic studies as well as high-resolution functional analysis of the parasite’s surface proteins and their interactions with mosquito cells. These assays could also be used to validate transmission-blocking drugs and vaccines.

## Advances, challenges, and opportunities in infection biology

The past 5 years have seen rapid progress in understanding the biology of *Plasmodium* infection in the human liver. Increased availability of primary human hepatocytes has allowed the development of multiple in vitro platforms, all tailored toward the concept of a miniaturized experimental liver model [28, 29, 124]. Importantly, these innovations have allowed the liver stages of infection to be fully recapitulated outside the human host for the first time [26, 125]. They have also spurred the development of reagents to explore the biology of sporozoite infectivity and liver stage development and provided the first glimpse of the *P*. *vivax* hypnozoite [26, 28].

In parallel, the development of humanized mouse models of *P*. *vivax* and *P*. *falciparum* infection have opened up the potential for surrogate in vivo models of human liver infection [26] and allowed the first genetic crosses of parasites (*P*. *falciparum*) outside of a primate [31]. Studies in primates continue to play an important role; the *P*. *cynomolgi* monkey model of liver infection is the only in vivo relapse model of the *P*. *vivax* hypnozoite [30, 32, 126]. Combined with controlled human malaria infections [34, 35, 38, 127, 128] and in vitro models, these tools have highlighted key differences in the biology of different parasite species (specifically, *P*. *vivax* and *P*. *falciparum*) and paved the way for understanding the cellular biology of liver infection and the immune response and for performing high-throughput drug candidate screening.

To facilitate efforts aimed at eradication, we have identified a number of transformative actions in the field of infection biology. A transformative innovation would be the in vitro cultivation of large numbers of infectious *P*. *falciparum* and *P*. *vivax* sporozoites, bypassing the mosquito vector. This would not only facilitate basic research but also contribute to whole-parasite vaccine development. Alternatively, advances in the preservation of sporozoite viability and infectivity after mosquito dissection and/or the engineering of mosquitoes to produce sporozoites at high levels would increase the availability and distribution of infectious material for research purposes.

Improved liver-stage cell lines could also have a transformative effect on the pace of novel drug and vaccine development, especially for *P*. *vivax* [28–30]. Cell lines provide readily available, immortal, and genetically identical cells, allowing researchers to reliably obtain the same sensitivity measurements for each compound or antibody. This development could enable high-throughput drug screening for discovery of liver stage-specific compounds targeting either parasite functions [129] or human targets necessary for parasite development. Moreover, the availability of robust and inexpensive in vitro hepatocyte infection models for *P*. *vivax* and *P*. *falciparum* may allow the development of better in vitro assays for antibody-dependent inhibition of invasion (akin to virus neutralization assays) and cell-mediated killing of infected cells. This could allow the discovery of human monoclonal antibodies with broadly neutralizing activity, whose cognate antigens could then be used to create vaccines that give sterilizing immunity. Recent advances in proteomics and mass spectrometry may also support the identification of biomarkers for exoerythrocytic stages that are relevant in vivo.

## Advances, challenges, and opportunities in asexual-stage biology

### Defining the parasite “resistome”

Notable advances in asexual biology over the past 5 years include improvements in functional genomics, such as more robust RNA sequencing methods [130–132], a deeper understanding of transcription factors such as activator protein 2 (ap2) transcription factors [133] or alternative RNA splicing [134], and whole genome sequencing and genotyping of both field isolates and evolved cultures (see Table 1). Due to its rapidly decreasing cost and increasing accuracy, sequencing has accelerated our understanding of the mechanisms and modes of action of current and new antimalarials through drug-resistant parasite selection in vitro (reviewed in [135]) as well as population genetics of the parasite in vivo [62, 136]. Although numerous studies have described using in vitro evolution and whole genome analysis to both find targets of new antimalarial compounds and identify genes conferring resistance [62, 137, 138], in most cases, only a handful of genes were identified. Now that single cell sequencing is becoming a reality [139], we are in a position to identify every gene (and potentially allele) that contributes to drug resistance, thus defining the parasite “resistome.” The complete genetic basis of parasite drug resistance should provide better molecular markers of whether parasites have acquired resistance to drugs that may be used in elimination campaigns, informing drug or drug combination selections (See malERA Refresh paper on resistance [140]).

### Systematic characterization of the asexual-stage parasite

The systematic knockout of genes in *P*. *berghei* has led to numerous advances in our understanding of fundamental asexual biology [141, 142], including the *P*. *berghei* identification of essential genes and pathways [143–146], greater understanding of merozoite invasion and egress [147–150], discovery of the parasite’s export machinery [145, 151, 152], and revealing how the red cell cytoplasm and membrane are remodelled [153, 154]. Such studies point to the critical nature of these processes and have opened the possibility of targeting them with drugs or vaccines.

Yet, major gaps remain in our knowledge of gene function in *P*. *falciparum* and, to an even greater extent, in other species (including *P*. *vivax*, *P*. *ovale*, and *P*. *malariae*) in which genetic diversity is also relatively uncharacterized. Although in many cases, genomic variants can be readily identified in sequencing data, poor annotations for predicted genes in the *P*. *falciparum* genome continue to slow progress. For example, we know little about the cellular function of the *pfkelch13* gene, a major contributor to artemisinin resistance ([62, 155, 156], reviewed in [67]). Given that it is more efficient and inexpensive for the community to work together to functionally annotate the *P*. *falciparum* genome systematically rather than in a 1-researcher-1-gene fashion, coordinated large-scale projects with a focus on the easily accessible *P*. *falciparum* asexual blood stage should be considered. Such systematic data would also help in the interpretation of whole genome sequencing data from drug- or vaccine-resistant parasites. Desirable genomic annotations include the location of key transcription factor binding sites, transcriptional start and stops sites [157], epigenetic chromatin modifications, and the cellular localization of encoded proteins. These consortium-acquired data are critical to predict whether genetic variants discovered through genome sequencing of model organisms and humans are indeed functional and could also help prioritize antigens for vaccine development. In addition, if better in vitro culture systems can be developed for *P*. *vivax* (see “Advances, challenges, and opportunities in transmission biology”), these systematic approaches could be extended to this important species. A potential model for such a consortium-based effort is the human ENCODE project, which has identified functional elements in the human genome [158].

### Using metabolomics to identify biomarkers and develop diagnostics

There have been major advances in the use of modern mass spectrometry-based methods for identifying and profiling metabolites from parasite-infected cells [159–161] as well as determining the mode of action of drugs through the metabolic perturbations of exposed parasites [162–165]. Two key areas in which metabolomics-based approaches have yet to make a significant impact are biomarkers and diagnostics. Given the difficulty and cost associated with identifying infected individuals (particularly those who are asymptomatic—see malERA Refresh paper on reservoir and transmission [166]), the development of effective metabolomic biomarkers with significant correlation to infection would represent a critical advance. Furthermore, to determine host markers of infection, field samples across a broad range of infectivities, including asymptomatic carriers, should be studied using metabolomic methods. Such analyses should also aim to span all *Plasmodium* parasite species as well, particularly *P*. *vivax*.

## The question of persistence: Where do parasites—And mosquitoes—Hide?

In the drive towards elimination and eradication, a key question is how and where malaria infection persists in both humans and mosquitoes, both in individuals as well as populations. Recent genomic studies indicate that parasites may also persist in an additional zoonotic reservoir in nonhuman primates [167–169], although how this contributes to disease transmission in humans is currently unclear.

Persistence of malaria occurs in 2 modalities—asymptomatic carriers and latent liver stages. The asymptomatic carriers represent a significant threat to the reintroduction of malaria; thus, the identification of such carriers requires a heightened level of awareness and detection. The absence of symptoms in an individual may reflect the presence of disease-prevention host responses in the absence of sterilizing immunity, thereby allowing persistent parasitemia or the sequestration of parasites in sites (e.g., the liver or bone marrow) in which they are “hidden” from the immune system. Understanding the relative contributions of both human immune responses and parasite biology will be essential to maximize the efficacy of antimalarial interventions, particularly vaccines.

Parasite persistence in the liver is a major hurdle for elimination efforts, particularly for *P*. *vivax*, because of its rapid development of gametocytes in humans, enabling transmission before the onset of clinical symptoms. Insights have emerged from studies of nonhuman primate models and humanized mouse models [26] in which parasite forms resembling hypnozoites demonstrated some biologic activity. These findings imply that sensitive technologies, such as proteomics and metabolomics, may identify markers likely secreted at these stages. Such markers would require field validation but ultimately could be incorporated into point-of-care diagnostics, eliminating the need for primaquine or tafenoquine in mass drug administration campaigns and informing epidemiological studies of the load of hypnozoite infection in endemic regions.

The transmission of *Plasmodium* infections with low or submicroscopic levels of circulating gametocytes suggests the possibility of nonrandom sequestration of gametocytes at sites in peripheral skin that are accessible to mosquitoes. *P*. *falciparum* gametocytes have recently been found to have an extended maturation period in the bone marrow [103, 170]. A clear implication of this observation, however, is that gametocytes detected in the peripheral circulation may not accurately reflect overall or infectious gametocyte levels and that more sensitive assays are needed to identify potential sources of transmission.

### Mosquito vector persistence

The aspects of vector biology that enable malaria persistence remain to be investigated and will be critical not only for informing and targeting current elimination and eradication strategies but also for the development and successful deployment of novel vector-based interventions. Recent data suggest that, in Africa, both mosquito estivation (dry season diapause) and long-distance migration contribute to the persistence of sub-Sahelian mosquito populations following a dry season, but in a species-specific manner [70]. New genomic resources have facilitated the understanding of fine-scale mosquito population structures [77, 171] suggesting large and stable populations [74–76]. The contribution of the observed genomic patterns to population persistence is unclear at this point, and a better understanding of the life history, ecology, and migration rates of vectors that result in the observed genomic patterns between populations is needed. Similar studies in non-African mosquito populations are needed.

Mosquitoes also persist through physiological resistance to insecticides (see malERA Refresh paper on resistance [140]), either through target site mutations, increased expression of detoxifying enzymes, or cuticular thickening. Genomic markers associated with resistance continue to be identified, yet together they do not adequately explain all the variation in insecticide resistance phenotypes observed in natural populations, and their relative functional impact in the field remains poorly understood.

Mosquito persistence may also occur due to heritable changes in behavior selected for by control interventions, so-called behavioral resistance. Recent work has captured mosquito interactions with bednets using mosquito-tracking cameras [172] and could be extended to other interventions (e.g., traps, sprays, repellents). Consistent longitudinal studies are also needed to track changes in mosquito biting behavior (e.g., outdoor versus indoor, evening versus night) after the use of interventions and to discriminate these changes from variation in species frequencies at specific sites. Subsequent genomic analyses could then reveal if there is a genetic component to these modified behaviors.

## Technology and its application to malaria biology

### Fundamental technologies: Genomics and transcriptomics

Whole genome sequencing has already had a major impact on multiple areas of parasite and vector research. It has transformed our understanding of parasite biology and drug resistance (see “Advances, challenges, and opportunities in asexual-stage biology”). In addition, it has been widely used to study the population genetics of mosquito species in the field [74–76, 173], and the genomes of 19 *Anopheles* species spanning 3 subgenera and including major and minor malaria vectors from diverse geographical locations have now been sequenced [77, 171]. These genomic resources have improved our understanding of the patterns of gene flow within and among mosquito populations. These “big data” resources available to the research community allow for powerful comparative functional and evolutionary analyses that will help elucidate the common basis of vector competence and identify effective vector control targets across multiple species. Recent work using these datasets has identified a reproductive trait with consequences for vectoral capacity that has evolved within the *Anopheles* genus and presents new potential targets to induce sterility in field populations [174–176]. Additional targets may be identified as our understanding of the biological coordination of simultaneous egg development and parasite transmission is improved. The declining cost of sequencing will make such studies more feasible in the future, such that a mosquito resistome—similar to the parasite resistome—may be compiled.

Further advances in genomic technology will enable a detailed analysis of natural populations of *Plasmodium spp*. at a worldwide scale. These include single cell technologies for genome sequencing and transcriptomic analyses, genotyping, and whole genome sequencing from dried blood spot samples. In addition, further comparative genomics [177] among all *Plasmodium* species infecting humans as well as those infecting nonhuman primates should identify key pathways in host switching. Genomic analysis of longitudinal samples will allow for the identification of population structure changes associated with changing epidemiology and emerging drug resistance. Coupled with gene-editing technologies, hypotheses generated by comparative genomics can be functionally tested.

Technical advances in RNA sequencing now make it feasible to interrogate the dynamic gene expression profiles of both the human host and the parasite during infection. This will provide new insights into the host response during infection and the potential adaptation of parasites during the infective process.

### Gene-manipulation technologies: Genome editing and transgenics

Genome engineering tools, such as CRISPR/Cas9 systems (see glossary in the malERA Refresh Introductory paper [178]), have transformed the ability to manipulate the genomes of *P*. *falciparum*, *P*. *berghei* (reviewed in [179]), and *Anopheles* and understand gene function. CRISPR/Cas9-based genetic engineering of *P*. *falciparum* asexual blood stages has allowed for more complex genetic modifications within the parasite; for example, the tetracycline repressor protein (TetR) aptamer system to control gene expression [84] utilized CRISPR/Cas9 as an initial step to introduce the aptameric cassette. Beyond CRISPR/Cas9, however, there have been several other successful gene-editing technologies (see Table 1).

With these powerful tools in place, we can now scale up the generation of conditional and/or complete knockout parasite libraries containing every single gene in the genome. Such an effort would greatly enhance our understanding of the biology of the parasite at all stages of development, as well as identify the functions of many hypothetical genes.

### Gene-manipulation technologies: Gene drives

Mirroring the advances in gene-editing capabilities in the parasite, *Anopheline spp*. genomes can also now be engineered with unprecedented precision (see Table 1). Recent reports show that CRISPR/Cas9 gene-editing tools can be used for the generation of gene-drive systems [91, 92] that manipulate genetic inheritance in mosquitoes to spread anti-*Plasmodium* transgenes (population modification/replacement strategies) or lethality-inducing transgenes (population suppression strategies) through natural mosquito populations. Mendelian inheritance predicts 50% of offspring will inherit a transgene carried on one of a parent’s chromosomes. Genetic drive is the increased transmission of a genetic element to over 50% of offspring so that it increases in frequency in each generation. A gene drive typically refers to an artificial transgene that shows genetic drive by giving it the ability to trigger its own replication. A gene-drive transgene is copied from one chromosome to its homologous chromosome within germ line cells. With both chromosomes carrying a copy of the transgene (a homozygous germ line), all sperm or eggs derived from these cells will also carry the transgene, and if copying occurs in all germ cells, 100% of offspring will inherit the gene drive. This allows rapid spread of the gene drive (and its anti-*Plasmodium* cargo) into the mosquito population. A valuable debate on the safe use of gene drive systems has begun within the scientific community [180].

The feasibility of using gene drive strategies for mosquito control will need additional research efforts in 3 key areas. First, an understanding of mosquito mating biology and the determinants of male mating success and female mate choice will need to be developed. Colonization is likely to impact the mating ability of species that exhibit such a complicated mating behavior as swarming; mating competitiveness will be a key determinant of gene drive success. Second, effective, “evolution proof” gene-drive systems should be generated to preempt the selection of mosquitoes that are resistant to the drive mechanisms, which would otherwise reduce the efficiency of the drive. Gene drives will need to be optimized by testing different gene-drive architectures, especially if CRISPR/Cas9 mechanisms prove problematic. Third, effective antimalarial genes will need to be evaluated in a reliable and reproducible manner; many anti-*Plasmodium* factors have been identified and should be systematically tested in laboratory conditions for their ability to block parasite development within the mosquito host.

Consideration should be given to the formation of a consortium to evaluate and prioritize promising transgenic strategies and test these in multiple anopheline species and against a number of *Plasmodium* isolates. This represents an opportunity to avoid duplication of work; however, we would also argue for head-to-head comparison of transgenic strategies. Such a consortium could centralize resources, particularly in developing transgenic mosquitoes (e.g., injection service, mail-order mutants) and potentially a mutant library, but, currently, the space required for mosquito-line maintenance prevents this. As forward and reverse genetic screens become more realistic, we should develop methods to cryopreserve mosquito lines or, more realistically, store plasmids for injection to recreate lines as needed.

### Cell- and tissue-based technologies

Since the discovery of malaria parasites by microscopy [181], imaging has played a central role in malaria research. However, recent advances in imaging techniques have allowed visualization of the parasite and its interactions with the mammalian host and insect vector at an unprecedented level of resolution [101] [182] [102]. We can expect that imaging will reveal other novel insights into the biology of human malaria parasites and play a major role in the science of malaria eradication.

### New technologies to support tool development: Biomarkers and novel diagnostics

As our understanding of parasite biology advances—including insights into sequestration and dormancy—the potential to leverage emerging technologies to support the discovery of biomarkers of infection (see above) increases. Such insights into parasite biology are laying the foundation for novel diagnostic approaches based on more sensitive techniques to detect parasite byproducts (e.g., hemozoin) [183] or volatile substances [184]. When noninvasive, rapid, and inexpensive, these diagnostic approaches are likely to facilitate the identification of infected individuals who may be asymptomatic and/or functioning as reservoirs (see malERA Refresh papers on Tools [185] and the Reservoir and Transmission [166]).

Exosomes are key new players implicated in intercellular communication without direct cellular contact [186] and have a potential role as biomarkers [187]. The release of microparticles is augmented in human malaria [188, 189], and exosomes containing parasite proteins have been shown to be produced by infected cells [190] as well as by parasites [191, 192].

### New technologies in vaccine development and leveraging existing human volunteer sample datasets

Protective immunity requires that human hosts recognize and respond appropriately to parasite-derived antigens and epitopes. Such immunity is complex, however, requiring both innate and acquired responses and biological regulation of such responses as well as ensuring the responses’ durability. Malaria parasites utilize a number of mechanisms to evade these immune responses, which infected hosts must then overcome. In this context, there is a fundamental gap in understanding the correlates of protective immunity in the human host that target exoerythrocytic-stage parasites in both *P*. *falciparum* and *P*. *vivax*. Multiple new technologies are now available to identify antigens and epitopes that are the targets of innate and acquired immune responses. Examples include high-throughput genomic sequencing, transcriptomics, and proteomics. Structural vaccinology [193–195] has proven immensely powerful in viral vaccine development through improved immunogen design and is now being applied to asexual blood stages [94–97]. Near-atomic resolution cryo-electron microscopy is now being used to inform antigen and drug target selection as well as the rational design of potent immunogens [196–198]. In addition, new technology platforms and novel adjuvants are being incorporated into vaccines to ensure appropriate immune responses are elicited. Approaches based on structural biology [98–100] and genomic sequencing [199] are now being introduced into the clinical evaluation of candidate malaria vaccines. These efforts provide an opportunity to further define the effective targets as well as the nature of protective immune responses.

An effective *P*. *vivax* vaccine strategy also needs to contend with the challenge of relapse infections. To prevent “relapse outbreaks,” antirelapse vaccines will need to be multistage and multivalent, including components to suppress blood-stage parasites emerging from the dormant liver stages as well as block transmission. There are relatively few *P*. *vivax* vaccine candidates progressing currently through the global pipeline [200].

Controlled human challenge studies are potentially transformational in enabling our understanding of the human immune response to malaria. Coupling controlled infections with technical advances for interrogating human immune cells in real time can give us new insights into both the temporal response and the contributions from innate and acquired immunity. Additionally, deeper interrogation of the immune profile of naturally acquired infections could also provide key insights. Providing access to them will require forethought in preparing future proposals, particularly with respect to human subject approvals, repository deposition, and community sharing. Harnessing available systems through existing networks as well as ongoing clinical trials could provide the necessary reagents and access to human samples.

### Drug design and screening

The identification of potential targets through metabolomics and systems biology approaches coupled with advances in structural biology is now facilitating the design of compounds likely to interact with such targets. Moreover, high-throughput screening technologies are facilitating more rapid identification and prioritization of compounds for further investigation as potential leads, though corresponding techniques in high-throughput synthesis and characterization of small molecules require further development. In a reverse approach, high-throughput phenotypic screens are also enabling the selection of compounds whose structures can subsequently be used to inform the identification of potential molecular interactions and metabolic pathways for further analysis as targets for pharmacologic intervention (reviewed in [201]). It is important to note that because malaria primarily affects the developing world, the opportunity for profit is reduced. Malaria, with the assistance of the community and funders such as Medicines for Malaria Venture (MMV), has and will continue to function as a model for open source drug discovery [202–204].

### Technologies targeting mosquito-based interventions: Paratransgenesis and genetically modified mosquitoes

Recent years have seen a focus toward the identification of microbial populations that can block parasite development in the mosquito vector [205–208]. Genetic modification of these bacterial populations (paratransgenesis) could be a key tool, particularly for the control of outdoor biting and resting mosquito populations that are not currently targeted by insecticide-based strategies. Advances in *Wolbachia* bacteria experiments in *Anopheles* mosquitoes are particularly promising. *Wolbachia* are intracellular endosymbiotic bacteria that, in some insects, spread through populations by maternal transmission and cytoplasmic incompatibility. These endosymbionts were shown to block malaria parasite development in artificial settings [209] and were negatively correlated with *Plasmodium* infections in natural *A*. *coluzzii* populations from Burkina Faso [210, 211]. Two key research priorities are the development of a method to transform *Wolbachia* to deliver effective antiplasmodial genes and understanding the role of natural *Wolbachia* infections in malaria transmission dynamics.

In light of widespread resistance to currently used insecticides, the identification of alternative, safe, active compounds that can extend the lifetime of long-lasting insecticide-treated nets (LLINs) and indoor residual spraying (IRS) is imperative. The study of key pathways in mosquito reproduction, susceptibility to infection, blood feeding behavior, and longevity that can be effectively targeted to reduce vectoral capacity is therefore a priority. For example, new sterilizing compounds that interfere with key hormonal reproductive pathways, such as those regulated by juvenile hormone and 20-hydroxyecdysone, could be incorporated into mosquito nets to reduce mosquito fertility, including insecticide-resistant mosquitoes that may survive exposure to the net.

A key issue in applying these novel strategies will be achieving effective colonization of anopheline species, as the lack of mosquito colonies is preventing studies on the biology of important malaria vectors. An important breakthrough has been the recent colonization of *A*. *darlingi*, the most important American vector [93]. On the road to eradication, a deeper understanding of the biology and behavior of these species will be essential.

## Conclusions

As illustrated above, recent advances in basic science are providing deeper insights into the biology of the parasite, the mosquito vector, and the human host as well as their interactions at molecular, cellular, and organismic levels. Coupling these insights with recent technologies that help pinpoint potential methods to intervene or disrupt essential interactions can spur the use of novel tools to help eliminate and, ultimately, eradicate malaria.

## Abbreviations

ap2: activator protein 2
CRISPR/Cas9: clustered regularly interspaced short palindromic repeats/associated protein-9 nuclease
dsRNA: double-stranded ribonucleic acid
ENCODE: Encyclopedia Of DNA Elements Project
GWAS: genome-wide association study
IRS: indoor residual spraying
LLIN: long-lasting insecticide-treated net
malERA: Malaria Eradication Research Agenda
MMV: Medicines for Malaria Venture
TetR: tetracycline repressor
